# Regularized Multi-View Subspace Clustering for Common Modules Across Cancer Stages

**DOI:** 10.3390/molecules23051016

**Published:** 2018-04-26

**Authors:** Enli Zhang, Xiaoke Ma

**Affiliations:** School of Computer Science and Technology, Xidian University, Xi’an 710071, Shaanxi, China; yuleeo@163.com

**Keywords:** conserved modules, network analysis, subspace clustering, regularization, protein interaction networks

## Abstract

Discovering the common modules that are co-expressed across various stages can lead to an improved understanding of the underlying molecular mechanisms of cancers. There is a shortage of efficient tools for integrative analysis of gene expression and protein interaction networks for discovering common modules associated with cancer progression. To address this issue, we propose a novel regularized multi-view subspace clustering (rMV-spc) algorithm to obtain a representation matrix for each stage and a joint representation matrix that balances the agreement across various stages. To avoid the heterogeneity of data, the protein interaction network is incorporated into the objective of rMV-spc via regularization. Based on the interior point algorithm, we solve the optimization problem to obtain the common modules. By using artificial networks, we demonstrate that the proposed algorithm outperforms state-of-the-art methods in terms of accuracy. Furthermore, the rMV-spc discovers common modules in breast cancer networks based on the breast data, and these modules serve as biomarkers to predict stages of breast cancer. The proposed model and algorithm effectively integrate heterogeneous data for dynamic modules.

## 1. Introduction

The advances in biological technologies, such as the RNA-seq, make it possible to generate genome-wide high-throughput data with various platforms. The world consortia, such as The Cancer Genome Atlas (TCGA) https://cancergenome.nih.gov/ and the Encyclopedia of DNA Elements (ENCODE) https://www.encodeproject.org/, have generated large-scale heterogeneous data on, for example, gene expression, DNA methylation, and mutation for various cancers or tissues (cells). The accumulated biological data provides a great opportunity to investigate the mechanisms of cancers.

Among these genomic data, great efforts have been devoted to the analysis of gene expression because regulation of gene expression refers to the control of the amount and timing of appearance of the functional product of a gene. Control of expression is vital to allow a cell to produce the gene products it needs when it needs them; in turn, this gives cells the flexibility to adapt to a variable environment, external signals, damage to the cell, and other stimuli [1,2,3]. The differentially expressed genes between two cohorts shed light on revealing the regulation mechanisms of cells. For example, Li et al. [4] demonstrated that PE1 inhibits stem cell self-renewal in human chronic myelocytic leukemia. To investigate the high-order relation among genes, network-based analysis has been devoted to gene expression, which extracts many interesting patterns that are different from differentially expressed genes. For instance, Langfelder et al. [5] proposed the weighted gene co-expression network analysis tool (WGCNA) to mine the co-expression modules.

Furthermore, biological networks have been proven to be powerful for describing and analyzing profile data, where each vertex represents a gene and each edge corresponds to an interaction between a pair of genes. There are many biological networks, such as gene regulation networks [6], signal transduction networks [7], protein–protein interaction (PPI) networks [8], disease networks [9], and gene regulation networks [10,11,12,13,14,15]. The accumulated biological networks provide an opportunity to explore the mechanisms of cells via mining the graph patterns. Great efforts have been devoted to network analysis, where the graph patterns shed light on the structure–function relations in biology. For example, Taylor et al. [16] analyzed the PPI network and demonstrated that the genes with large degrees (hub genes) play a critical role in the prognosis of breast cancer. Furthermore, Chuang et al. [17] showed that the pathways where genes are differentially expressed between two cohorts of cancer patients serve as biomarkers for predicting cancer metastasis.

However, a vast majority of analysis ignores the dynamics of data. Complex diseases, such as cancers, are dynamic and involve a continuum of molecular events associated with disease progression, from early warning events to catastrophic end-stage events [18]. How to extract modules associated with cancer progression is critical for discovering the mechanisms of cancers because these patterns provide clues for biologists for further research [19,20]. However, it is non-trivial to detect dynamic modules associated with cancer progression because it is difficult to characterize and extract dynamics of modules. Thus, the available algorithms for the dynamic modules differ greatly in terms of how to define dynamic modules and the strategies to discover the predefined patterns. Ma et al. [21] designed the *M-Module* algorithm to the common modules across various stages of breast cancer, and demonstrated that the dynamics of interaction strength is critical for the acceleration of heart failure [22]. Similar efforts have also been devoted to common and specific modules for breast cancer [23,24]. However, these algorithms only focus on extracting the common and specific modules associated with cancer progression. In [25], the authors developed the *NMF-DM* algorithm to investigate how the pathway dynamically recruits genes, for example, in cancer progression.

However, these algorithms are only based on gene expression or DNA methylation data and do not integrate any other data. In fact, integrative analysis of omic data has been extensively studied since it identifies interesting patterns that cannot be obtained by analysis of a single type of data [26]. Compared to the gene co-expression network, the protein interaction network is more reliable since the large co-expression value between a pair of genes does not imply physical interaction. Thus, the protein interaction network should be integrated with gene expression data to extract dynamic modules. Even though many algorithms have been developed to integrate protein interaction and gene expression data, no attempt has been made to identify modules associated with cancer progression. The reason is that the integrative analysis of these data is difficult because it involves both the breast progression and heterogeneity of data.

**Figure 1:**
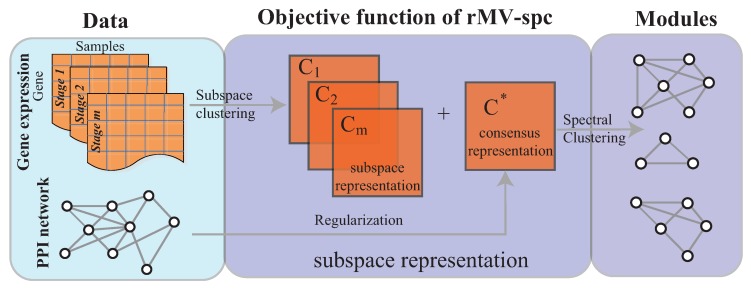
Overview of the rMV-s2c algorithm, which comprises two major components, namely, the regularized subspace clustering procedure, which obtains the subspaces for gene expression data of each clinical stage by regularizing the protein interaction network, and the module discovery procedure, which identifies common communities across cancer stages based on the consensus space.

In this study, we address the integration of gene expression data and a protein interaction network to mine the dynamic modules associated with cancer progression. As done in [21,22], the dynamic modules are defined as common modules that are co-expressed across various stages. To analyze cancer gene expression data, we adopt the multi-view subspace clustering algorithm with sparsity constraints to obtain a representation matrix for each view and a consensus matrix, as shown in Figure 1 (Supplementary Materials). By effectively integrating the protein interaction networks, we expected that the joint representation matrix *C* would not only balance the agreement across various stages but also preserve the topological structure of the protein interaction network. Therefore, the protein interaction network was incorporated into multi-view subspace clustering via regularization. In this way, the common module detection problem is transformed into a convex optimization. The interior point algorithm was used for convex optimization. The experimental results demonstrate that the proposed algorithm is more accurate than the state of the art. The modules obtained by our algorithm are more enriched by the known pathways and serve as biomarkers to predict cancer stages.

The rest of the paper is organized as follows: Section 2 proposes the mathematical model and algorithm. The related materials are presented in Section 3. The experimental results are provided in Section 4. The conclusion is discussed in Section 5.

## 2. Methods

The objective function and optimization procedure of the proposed algorithm, and the algorithm analysis, are presented in this section. The rMV-spc algorithm comprises two major components as shown in Figure 1.

### 2.1. Preliminaries

Prior to giving the detailed description of the procedure of rMV-spc, let us introduce some terminologies that are widely used in the forthcoming sections.

The protein interaction network can be modeled by an unweighted and undirected graph G=(V,E), where the vertex set $V=\{v_{1},v_{2},\ldots ,v_{n}\}$ contains all the genes (proteins) and the edge set $E=\{\left(v_{i},v_{j}\right)\}$ denotes the interaction between a pair of genes. The protein interaction network *G* can be represented by an n×n adjacency matrix *A*, where $a_{ij}$ =1 if vertex $v_{i}$ and $v_{j}$ are connected, 0 otherwise. The degree of vertex $v_{i}$ is the number of edges connected to it, i.e., $d_{i}=\sum_{j}a_{ij}$. The degree matrix *D* is the diagonal matrix with a degree sequence of *G*, i.e., $D=diag(d_{1},\ldots ,d_{n})$. The trace of a matrix *W* is the sum of diagonal elements of *W*, i.e., $trace\left(W\right)=\sum_{i}w_{ij}$.

Let {1,2,…,m} be a finite set of cancer clinical stages and the attached subscript *s* be the value of the variable at the *s*-th stage. The gene expression for cancer with various clinical stages $X=\{X_{1},X_{2},\ldots ,X_{m}\}$, where each $X_{i}$ is the gene expression for the stage *S*. The gene expression data $X_{s}$ is an $n_{s}\times n$ matrix, where each row corresponds a gene, each column represents a sample (patient), and element $x_{ijs}$ denotes the expression level of the *j*-th patients in the *i*-th gene at stage *s*.

### 2.2. Procedure of Algorithm

In the single-view clustering, the sparse subspace clustering (SSC) [27,28] represents each data point using a small number of data points from its own subspace. Given the data *X*, it amounts to the minimization problem as

(1) $$\min_{C}{∥C∥}_{1},\;s.t.\;X=XC,\;diag\left(C\right)=0$$

where ${∥C∥}_{1}$ is the $l_{1}$ norm, and constraint diag(C)=0 is used to avoid trivial solutions where a data point is represented as a linear combination of itself. In the case of the corrupted data, the above equation can be re-written as

(2) $$\min_{C}{∥C∥}_{1}+\frac{\lambda_{z}}{2}{∥Z∥}_{F}^{2},s.t.\;X=XC+Z,\;diag\left(C\right)=0$$

where the $l_{1}$ norm promotes sparsity of the columns of *C*, while the Frobenius norm favors small entries in the columns of *Z*.

Given gene expression associated with cancer progression $X=\{X_{1},X_{2},\ldots ,X_{m}\}$, the multi-view clustering finds representation matrices $C_{1},\ldots ,C_{m}$ across different stages and a joint representation matrix *C* that balance the agreement across various stages [29]. According to [30], we use the centroid based strategy to obtain the consensus matrix *C* for the subspace clustering. Therefore, Equation (2) becomes

(3) $$\begin{array}{cc} \min_{C_{1},\ldots ,C_{m},C} & \sum_{s=1}^{m}∥C_{s}{∥}_{1}+\frac{\lambda_{z}}{2}∥Z_{s}{∥}_{F}^{2}+\frac{\lambda_{c}}{2}{∥C_{s}-C∥}^{2}\\ s.t. & X_{s}=X_{s}C_{s}+Z_{s},\;diag\left(C_{s}\right)=0,s=1,\ldots ,m. \end{array}$$

We present the regularized multi-view sparse subspace clustering (rMV-spc) algorithm to discover the common modules in multiple views of gene expression for cancers. However, the common modules solely based on gene expression data assume that the genes within a module are co-expressed. In fact, protein interactions between genes are more reliable than the co-expression relation. Thus, it is promising to integrate the gene expression and protein interaction network to discover the common modules across cancer stages. However, the protein interaction network is sparse. Therefore, we also expect that the joint representation matrix *C* not only balances the agreement across various stages but also preserves the topological structure of protein interaction network *G*. According to [31], the local-structure-preserved embedding can be formulated as the trace form, which is defined as

(4) $$O\left(C,G\right)=Trace\left(C^{\prime }L_{G}C\right)$$

where $L_{G}$ is the Laplacian matrix of graph *G*, i.e., $L^{G}=D-A$. By imposing the topology preserving constraint, the model in Equation (3) is formulated as

(5) $$\begin{array}{cc} \min_{C_{1},\ldots ,C_{m},C} & \sum_{s=1}^{m}∥C_{s}{∥}_{1}+\frac{\lambda_{z}}{2}∥Z_{s}{∥}_{F}^{2}+\frac{\lambda_{c}}{2}{∥C_{s}-C∥}^{2}+\lambda_{G}Trace\left(C^{\prime }L_{G}C\right)\\ s.t. & X_{s}=X_{s}C_{s}+Z_{s},\;diag\left(C_{s}\right)=0,s=1,\ldots ,m. \end{array}$$

To solve the model in Equation (5), we adopt an alternative two-step procedure. Specifically, we update $C_{i}\left(1\leq i\leq m\right)$ by fixing *C*, while we update *C* by fixing $C_{i}\left(1\leq i\leq m\right)$. In each procedure, the problem in Equation (5) is a convex optimization, which can be solved using the convex programming algorithms [32,33], and the sparsity of solutions is also preferred [34,35]. In this study, we adopt the interior-point algorithm [32] to obtain matrix *C*.

After obtaining the consensus matrix *C*, we construct the affinity matrix *W* as

(6) $$W=C+C^{\prime }.$$

The spectral clustering algorithm is used to obtain the final modules. The procedure is depicted in Algorithm 1.

**Algorithm 1** The rMV-spc algorithm**Input:**  X: Gene expression data   G=(V,E): Protein interaction network **Output:**  ${\left\{V_{i}\right\}}_{i=1}^{k}$: Common modules 1:Update $C_{s}$ by fixing *C* and $C_{i}\left(i\neq s\right)$ based on the interior point algorithm [32] 2:Update *C* by fixing $C_{s}\left(1\leq m\right)$ based on the interior point algorithm [32]; 3:Normalize the columns of consensus matrix *C*; 4:Construct the affinity matrix $W=C+C^{\prime }$;5:Apply spectral clustering to obtain modules based on matrix *W*; 6:**return** common modules.

## 3. Materials

### 3.1. Statistical Significance of Modules

The statistical significance of common modules is computed based on the null score distribution of modules generated using randomized permutation. Each gene expression is completely randomized 1000 times by sample shuffling. The average Pearson coefficient among the gene pair with the module is used as the module score. To construct the null distribution for module scores, we perform the proposed algorithm on the randomized gene expression data. Using the null distribution, the empirical *p*-value of a module is calculated as the probability of the module having the observed score or greater by chance. *p*-values are corrected for multiple testing using the method of Benjamini–Hochberg [36]. An adjusted *p*-value of 0.05 is considered as significant.

### 3.2. Module-Based Features for a Support Vector Machine (SVM)

Given a module *C*, we normalize the expression level of each gene across all samples using z-score transformation [17], denoted by $Exp_{ij}$ for the *i*-th gene and *j*-th patient. For each sample *j*, the activity score of the *k*-th module is defined as the average gene expression of all genes within the module, i.e.,

(7) $$e_{C}=\sum_{i\in C}Exp_{ij}/\sqrt{\left|C\right|}$$

where |C| is the number of genes in *C*. For each patient sample, a feature vector is constructed by all modules.

### 3.3. Normalized Mutual Information

The normalized mutual information (NMI) [37] is based on the confusion matrix *N* whose rows correspond to the real modules in standard partition $P^{*}$ and the columns correspond to the modules in obtained partition *P*. The element $N_{ij}$ is the number of vertices overlapped by the *i*-th real and *j*-th predicted module. The NMI is defined as

$$NMI\left(P,P^{*}\right)=\frac{-2\sum_{i=1}^{\left|P\right|}\sum_{j=1}^{|P^{*}|}N_{ij}\log\left(\frac{N_{ij}N}{N_{i.}N_{.j}}\right)}{\sum_{i=1}^{\left|P\right|}N_{i.}\log\left(\frac{N_{i.}}{N}\right)+\sum_{i=1}^{|P^{*}|}N_{.j}\log\left(\frac{N_{.j}}{N}\right)}$$

where |P| is the number of modules in *P* and $N_{i.}$ is the sum of the *i*-th row of the matrix.

### 3.4. Artificial Networks

The GN benchmark network, where each network consists of 128 nodes that are grouped into 4 clusters of equal sizes, is introduced in [38]. Every node has an average degree of 16 and shares $Z_{out}$ edges connecting nodes outside of the module to which it belongs. As parameter $Z_{out}$ increases from 1 to 8, the detection of clusters in the networks becomes increasingly difficult. In this study, we combine three GN networks to construct the artificial networks to testify the performance of the proposed algorithms, where the first two networks are used for the multiple views and the last network is used for the regularization.

### 3.5. Breast Cancer Gene Expression Data

The gene expression data for breast cancer is downloaded from the TCGA Data Portal, where the clinical stage information for patients is also available. The RPKM values (RNA-seq IlluminaHiSeq_RNASeq with level 3) are used. There are 809 samples across four stages (Stage I: 129, Stage II: 458, Stage III: 209, Stage IV: 13).

### 3.6. Protein Interaction Network

The protein interaction network is downloaded from BioGrid database https://thebiogrid.org/, which comprises 22,365 proteins (genes) and 437,751 interactions among genes. There are 435,543 physical interaction and 2208 genetic interactions.

## 4. Results

To validate the performance of the proposed algorithm, three state-of-the-art algorithms are selected to make a comparison of both artificial data and breast cancer data. The compared algorithms are the M-Module algorithm [21], multi-view clustering (MV-NMF) [39], and spectral clustering [40]. Notice that the spectral clustering cannot be applied to the multiple networks directly. Thus, we apply the spectral clustering to each network and then combine the results on each network based on consensus clustering (CSC).

Two types of datasets, including both the artificial and real breast cancer data, are employed for a comparison between various algorithms. The artificial networks are adopted to test the accuracy of the rMV-spc algorithm, and the breast cancer data are used to determine the applicability of the proposed algorithm in discovering common modules in real networks with strong backgrounds.

### 4.1. Benchmarking Performance on the Artificial Networks

In the artificial networks, we combine three GN networks, where the first two networks are used for multiple views and the remaining one is used for regularization (Materials). To increase the difficulty in discovering the common modules, we increase the parameter $Z_{out}$ from 1 to 8 while we fix $Z_{out}$ as 6. To quantify the performance of algorithms, the normalized mutual information (NMI) is adopted since the community structure is known in the artificial networks (Materials).

**Figure 2:**
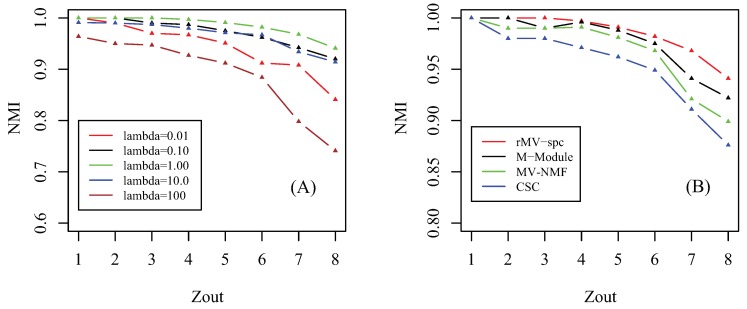
Parameter effect and performance of the compared algorithms on artificial data. (**A**) Parameter effect: how the NMI changes as parameter λ increases from $10^{-2}$ to $10^{2}$. (**B**) Performance as a function of the amount of parameter $Z_{out}$ in the simulated data among various algorithms, where NMI is used as the performance measure.

Prior to giving the performance of algorithms, we first investigate how the parameter affects the performance of the proposed algorithm. Notice that there are three involved parameters: parameter $\lambda_{Z}$ controls the importance of the regularizer of factorization, parameter $\lambda_{C}$ determines the tradeoff between the consensus matrix among multiple views, and parameter $\lambda_{G}$ denotes the importance of the network for regularization. Similar to [41], we assume that these parameters are equal since we hypothesize that all items for regularization are equally important. By setting parameter $\lambda \in \{10^{-2},10^{-1},10^{0},10^{1},10^{2}\}$, we check how the accuracy of the proposed algorithm changes as parameter $Z_{out}$ increases from 1 to 8 in terms of NMI, which is shown in Figure 2A. As λ increases from $10^{-2}$ to $10^{0}$, the accuracy of the rMV-spc algorithm increases and achieves the best performance at λ = 1. The reason is that, when λ is small, the objective function is denominated by subspace clustering, and the contribution of items of regularization is subtle. As λ increases, the contribution of regularized items becomes increasingly important, which improves the accuracy of rMV-spc. As λ increases from $10^{0}$ to $10^{2}$, the accuracy of the proposed algorithm decreases dramatically. The reason is that, as lambda continues to increasing, the objective function of rMV-spc is dominated by the regularization, resulting in the decrease in the performance of the algorithm. Furthermore, the proposed algorithm is robust since its accuracy is stable for a wide range of λ values. In all experiments, we set λ = 1.

We compare the MV-NMF, CSC, M-Module, and rMV-spc algorithms on the artificial networks in terms of accuracy, which is shown in Figure 2B. From the panel, we assert that the proposed algorithm achieves the best performance, followed by M-Module, MV-NMF, and CSC. While the M-Module is inferior to the rMV-spc algorithm, it is much better than the others. There are two possible reasons why the proposed algorithm outperforms the other methods. First, the subspaces are more precise in characterizing the module structure in multiple view data compared with the data in the original space. Second, the proposed algorithm incorporates both the subspace and topological information, which provides a better way to characterize the structure of common modules. Moreover, it is easy to conclude that the performance of algorithms decreases dramatically as $Z_{out}$ increases from 1 to 8 because the module structure becomes fuzzy as $Z_{out}$ increases. For example, the NMI is about 1 when $Z_{out}\leq 4$. As $Z_{out}>4$, the NMI value decreases dramatically.

### 4.2. Benchmarking Performance on the Breast Cancer Networks

The artificial data is used to test the performance of the proposed algorithm in detecting the common modules in terms of accuracy. To check whether the proposed algorithm can identify common modules across various clinical stages in the data with biological background.

**Figure 3:**
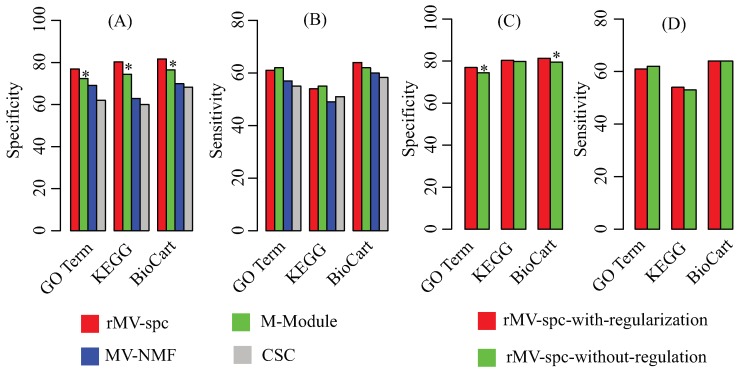
Performance of the compared algorithms on the TCGA breast cancer data. (**A**) Specificity of modules obtained by various algorithms in the known pathway enrichment analysis of various algorithms. (**B**) Sensitivity of communities obtained by various algorithms in the known pathway enrichment analysis of different algorithms. (**C**) Specificity of modules obtained by the proposed algorithms with and without the regularization of the protein interaction network. (**D**) Sensitivity of modules obtained by the proposed algorithms with and without the regularization of the protein interaction network. The * denotes that the difference is significant using Fisher’s exact test with a cutoff of 0.05.

Because the true modules are unknown, multiple reference pathway annotations, including Gene Ontology [42], KEGG [43], and Biocart [44], are used to determine the effectiveness of the algorithms by using the enrichment analysis (Materials). To evaluate the performance, we use specificity and sensitivity to quantify the accuracy, where specificity is defined as the fraction of the predicted modules that significantly overlaps with at least one reference pathway, while sensitivity is defined as the fraction of the reference pathways that significantly overlaps with at least one predicted module. Figure 3A,B shows that the rMV-spc algorithm achieves higher specificity while maintaining comparable sensitivity than the other methods. Specifically, the specificity values of rMV-spc are 76.9%, 80.3%, and 81.7% for the GO, KEGG, and BioCart pathways, respectively, while those of the M-Module algorithm are 72.4%, 74.4% and 76.5%. The results demonstrate that the common modules obtained bythe proposed method are more enriched by the known pathways than those obtained by others. Notice that the rMV-spc algorithm is inferior to M-Module in terms of sensitivity. We check the significance of the difference between rMV-spc and M-Module on sensitivity using the Fisher exact test with a cutoff of 0.05. The results demonstrate that the difference in specificity is significant, while it is not significant in terms of sensitivity.

The proposed algorithm integrate both the gene expression and protein interaction networks. Then, we ask what is the different if the protein interaction network is not integrated. The specificity and sensitivity of modules are shown in Figure 3C,D. From the panel, we assert that the integration of the protein interaction network increases the percentage of modules that are enriched by known pathways. The results demonstrate that the integration is promising in identifying the common modules associated with cancer progression.

### 4.3. Common Modules Serve as Biomarkers to Predict Breast Cancer Stages

It has been shown that the hub genes [16] and modules [17,21] are predictive for the breast cancer diagnosis. Thus, we hypothesize that the common modules can also be used to predict the stages of breast cancer. Following [17], we construct module-based features to predict the stages of breast cancer (Materials). For each module, we construct a feature vector that is the average of the gene expression of the genes within the modules. Based on the feature vectors, we use the SVM to predict the stage of cancers.

**Figure 4:**
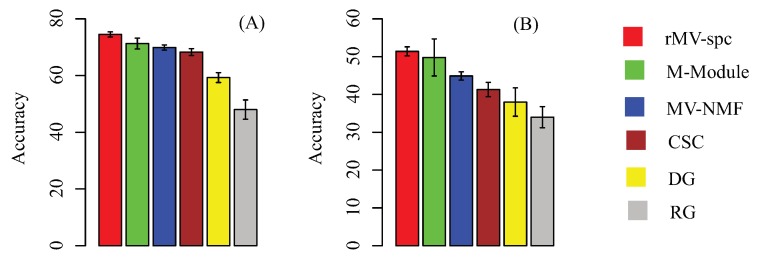
Subtype-specific methylation modules improve the accuracy of breast cancer stage classification using 50 independent 5-fold cross validations. (**A**) Classification accuracy of breast cancer stages using different feature sets, including the stage-specific modules obtained by various algorithms. Accuracy is defined as the number of patient samples correctly classified. The *Y*-axis is the accuracy and the error bar is for the standard deviation. (**B**) External validation by training on TCGA data and testing on the external data.

For a baseline comparison, we compare the classification accuracy by using the following feature sets: modules generated by other algorithms, size-matched differentially expressed genes, and randomly selected genes. We trained the support vector machine (SVM) classifier to perform multi-class classification. This SVM employed accuracy (the percentage of patients that are corrected classified) to measure performance. The results on the TCGA breast cancer data using five-fold cross validation are presented in Figure 4A. The modules obtained by our algorithms are more discriminative than the others. Specifically, the rMV-spc algorithm has significantly higher accuracy than the M-Module (74.5% vs. 71.3%). These results demonstrate that the common modules obtained by rMV-spc capture the specificity of pathways as breast cancer progression.

To further validate the performance of various algorithms, we evaluated the performance of the SVM classifiers by using external data (GSE5874). We trained the SVM classifier on the TCGA data and tested it on an external microarray dataset. Consistent results indicate that the performance is not due to hidden confounding factors in the TCGA dataset (Figure 4B). The accuracy of rMV-spc is 51.4%, while the accuracies of the M-Module, MV-NMF, CSC, and DGis are 49.8%, 44.9%, 41.3%, and 38.7%, respectively. The results show that the proposed algorithm is better than the available approaches in discovering common modules in data integration.

## 5. Conclusions

The advances in biological technologies enable the possibility of generating multiple genomic profiling of biological samples for various conditions. How to integrate the heterogeneous genomic data to extract patterns is critical since these patterns may shed light on the mechanisms of cancers. Even though many algorithms have been devoted to the integrative analysis of omic data, few attempts have been made to simultaneously integrate heterogeneous and time-series gene expression data.

In order to attack this issue, we provide a novel algorithm by considering the time and heterogeneity factors at the same time. In this study, the gene expression associated with cancer progression are projected to subspaces based on subspace clustering. In order to incorporate the protein interaction network, we treat it as a regularizer with an immediate purpose to alleviate the effects of heterogeneity. The experimental results demonstrate that the proposed algorithm is promising in discovering common modules across various cancer stages. We see ample opportunities to improve on the basic concept of rMV-spc in future work. For example, we can extend the algorithm by integrating more heterogeneous data, such as DNA copy number variation and methylation.

## Supplementary Materials

The following are available online.
